# Review: Miglitol has potential as a therapeutic drug against obesity

**DOI:** 10.1186/s12986-015-0048-8

**Published:** 2015-12-01

**Authors:** Satoru Sugimoto, Hisakazu Nakajima, Kitaro Kosaka, Hajime Hosoi

**Affiliations:** Department of Pediatrics, Ayabe Municipal Hospital, 20-1 Otsuka, Aono-cho, Ayabe city, 623-0011 Kyoto Japan; Department of Pediatrics, Graduate School of Medical Science, Kyoto Prefectural University of Medicine, 465-Kajiicho, Hirokoji-Kawaramachi, Kamikyo-ku, Kyoto, 602-8566 Kyoto Japan

**Keywords:** Miglitol, Obesity, Brown adipose tissue, Incretin hormones, Bile acid metabolism

## Abstract

The number of obese patients has increased annually worldwide. Therefore, there is a strong need to develop a new effective and safe anti-obesity drug. Miglitol is an alpha-glucosidase inhibitor (αGI) that is commonly used as an anti-diabetic drug, and there is growing evidence that it also has anti-obesity effects. Miglitol has been shown to reduce body weight and ameliorate insulin resistance in both clinical trials with adult patients and in rodent models of obesity. Although the specific mechanism of action of this effect remains unclear, some mechanisms have been suggested through experimental results. Miglitol has been shown to inhibit adipogenesis of white adipocytes *in vitro*, activate brown adipose tissue (BAT) in mice, influence bile acid metabolism in mice, and regulate the secretion of incretin hormones in humans. Among these results, we consider that BAT activation is likely the definitive mediator of miglitol’s anti-obesity effect. A unique advantage of miglitol is that it is already used as an anti-diabetic drug with no severe side effects, whereas many of the anti-obesity drugs developed to date have been withdrawn because of their severe side effects. Miglitol is currently used clinically in a limited number of countries. In this review, we provide an overview of the state of research on miglitol for obesity treatment, emphasizing that it warrants more detailed attention. Overall, we demonstrate that miglitol shows good potential as a therapeutic for the treatment of obesity. Thus, we believe that further investigations of how it exerts its anti-obesity effect will likely contribute to the development of a new class of safe and effective drugs against obesity.

## Background

Obesity is a worldwide health problem that causes various diseases such as diabetes, cardiovascular disease, stroke, and cancer. Many pharmaceutical companies have invested substantial capital and labor to develop anti-obesity drugs; however, most of the anti-obesity drugs that have thus far been approved and marketed have ultimately been withdrawn because of their serious adverse effects (e.g., psychological symptoms, increased risk of heart attack and stroke, and sudden death) [1, 2]. Miglitol is commonly prescribed to diabetic patients, as it reduces postprandial hyperglycemia by inhibiting alpha-glucosidase in the small intestine, and thereby prolongs carbohydrate absorption [3]. Miglitol was approved as an anti-diabetic drug in 1996 and has since been sold in Japan, the USA, Australia, France, Germany, Spain, Switzerland, and Mexico. Furthermore, there is growing evidence that miglitol also exerts an anti-obesity effect based on both animal and human studies. This review summarizes the basic and clinical research conducted to date on the anti-obesity effect of miglitol. We first provide an overview of recent and current clinical trials conducted for miglitol, given that the drug has long been used in a clinical setting for diabetes, and thus its effects on patients and potential anti-obesity potential were first observed in a clinical context. These observations and trials have motivated basic research studies conducted *in vitro* and with animal models to elucidate the underlying mechanisms of miglitol’s observed clinical effects. These studies are therefore described in the latter part of the review, demonstrating what is known and what remains to be discovered. Finally, we discuss the future directions of miglitol and its unique advantages for translation from an anti-diabetic to an anti-obesity drug, and compare its effects and potential to other currently Food and Drug Administration (FDA)-approved anti-obesity drugs. Collectively, this review highlights the need for further basic research as well as clinical studies in countries other than Japan, especially those with a high incidence of obesity.

### Clinical trials for miglitol’s anti-obesity effect

There have been four clinical trials (randomized open-label studies) reported to date, which have all been carried out in Japan, clearly demonstrating that miglitol shows an anti-obesity effect [4–7] (Table 1). In all four studies, miglitol significantly reduced the body weight and body mass index of obese or type 2 diabetic patients. Shimabukuro et al. [4] enrolled the largest number of patients (*n* = 111), which were divided into a life-style modification (LSM)-only group and an LSM-plus-miglitol treatment group. They demonstrated that miglitol lowered homeostatic model assessment-insulin resistance (HOMA-R) and decreased insulin levels during the oral glucose tolerance test, which suggested that miglitol could ameliorate insulin resistance. Subcutaneous/visceral fat mass and systolic/diastolic blood pressure were decreased in the LSM-plus-miglitol group. Furthermore, miglitol improved total cholesterol (T-Cho), low-density lipoprotein (LDL)-cholesterol, γ-glutamyl transpeptidase, high-sensitive C-reactive protein, and adiponectin in the blood. Mikada et al. [6] demonstrated that miglitol reduced total body fat mass and lowered systolic blood pressure. Furthermore, Narita et al. [5] and Sugihara et al. [7] demonstrated that miglitol was more effective than other alpha-glucosidase inhibitors (αGIs; e.g., voglibose or acarbose) at reducing body weight gain. No severe side effects were observed in any of these studies. There is further evidence that miglitol could reduce appetite and food intake in humans [8, 9], thus supporting its anti-obesity effect, although the evaluations in these studies were of a very short duration and were somewhat limited to derive a conclusive result.

**Table 1:** List of clinical trials examining migitol’s anti-obesity effect

Reference (Author, year, country)	Design and duration of intervention	Study participants	Comparison	Change of BW (kg) Mean ± SD	Results	Side effects
[4] Shimabukuro et al., 2012, Japan	Open-label, randomized-control. 12 weeks	111 drug-naive patients. Men and women aged 34–69 years with metabolic syndrome	Lifestyle modification (LSF) (*n* = 56) vs LSF with miglitol (*n* = 55)	Before/ After treatment :72.6 ± 11.7/ 68.9 ± 10.4	Parameters improved in LSF + miglitol: BW, systolic and diastolic blood pressure, HOMA-R, blood examination (T-cho, LDL, TG, γGTP, high sensitive CRP, HbA1c, 1,5-AG), insulin and blood glucose during OGTT, SFA , mean % change from baseline in VFA. Parameters improved in both groups: BMI, waist circumstance, VFA	Mild flatulence, abdominal pain, and diarrhea
[5] Narita et al., 2012, Japan	Open-label, randomized parallel controlled. 12 weeks	50 patients with type 2 DM with diet therapy alone or with oral hypoglycemic agents other than αGI	Miglitol (*n* = 26) vs voglibose (*n* = 24)	64.5 ± 14.0 /63.6 ± 14.0	Parameters improved in miglitol group: BW, BMI Parameters improved in both groups: HbA1c, 2-hour MTT (plasma glucose AUC, insulin AUC) Both miglitol and voglibose decreased total GIP and increased active GLP1 during the MTT (GIP was lower in the miglitol group than in the voglibose group).	Not documented
[6] Mikada et al., 2014, Japan	Open-label, randomized parallel, three armed. 24 weeks	41 patients with type 2 DM and overweight (BMI ≥ 25) aged 20–80 years	Miglitol (*n* = 14) vs sitagliptin (*n* = 14) vs both drugs (*n* = 13)	81.4 ± 11.2 /79.9 ± 11.5	(Data are shown for only the miglitol-treated group): BW, BMI, total body fat mass, systolic blood pressure, blood glucose-iAUC, and insulin-iAUC decreased after treatment compared with before treatment. Miglitol decreased total GIP-iAUC and increased total GLP1-iAUC, but did not affect active GIP-iAUC and active GLP1-iAUC.	Not documented
[7] Sugihara et al., 2014, Japan	Open-label randomized. 12 weeks	81 patients with obesity and type 2 diabetes (BMI ≥ 25) aged ≥40 years	Control (*n* = 22), miglitol (*n* = 18), acarbose (*n* = 22), voglibose (*n* = 19)	69.0 ± 11.2 /67.8 ± 11.2	In only the miglitol group BW and BMI decreased after treatment compared with before treatment at 4, 8, and 12 weeks. HbA1c decreased after treatment compared with before treatment in the control (at 12 weeks) and in the miglitol-treated group (at 4, 8, and 12 weeks).	Some digestive symptoms observed in the three αGI-treated groups

*Abbreviations*: *DM* diabetes mellitus, *BW* body weight, *HOMA-R* homeostatic model assessment-insulin resistance, *T-Cho* total cholesterol, *LDL* low-density lipoprotein, *TG* triglycerides, *CRP* C-reactive protein, *HbA1c* hemoglobin A1c, *1,5-AG* 1,5-anhydroglucitol, *OGTT* oral glucose tolerance test, *SFA* subcutaneous fat area, *VFA* visceral fat area, *BMI* body mass index, *MTT* meal tolerance test, *AUC* area under the curve, *GIP* glucose-dependent insulinotropic peptide, *GLP1* glucagon-like peptide 1, *iAUC* incremental area under the curve from 0 min during the 2-hour meal tolerance test

These four clinical trials conducted in Japan strongly suggest that miglitol exerts an anti-obesity effect in humans. Nevertheless, higher quality clinical research should be performed before miglitol can be conclusively demonstrated to reduce obesity in humans. Furthermore, these previous studies involved a small number of only Japanese patients; therefore, future studies should enroll a larger number of patients, and the studies should be carried out for a longer duration in a non-blinded manner. The studies should also be performed in countries that have a high rate of obesity.

Two of the four clinical trials described above tried to verify the mechanism by which miglitol reduces obesity [5, 6]. Narita et al. [5] and Mikada et al. [6] focused on the role of incretin hormones. Incretin hormones are secreted from the gastrointestinal tract in response to meals, and contribute to the regulation of glucose homeostasis by stimulating insulin secretion from the pancreas in a glucose-dependent manner [10]. Incretin hormones also have extra-pancreatic effects, including an anti-obesity effect. Glucose-dependent insulinotropic peptide (GIP) and glucagon-like peptide 1 (GLP1) are the two main incretin hormones. GIP promotes fat accumulation [11], whereas GLP1 reduces appetite and food intake [12–14]. These findings suggest that a strategy aimed at enhancement of GLP1 signals or suppression of GIP1 signals has potential to alleviate obesity. Narita et al. [5] demonstrated that miglitol decreased plasma GIP levels and increased plasma GLP1 levels. Specifically, they found that body weight change was correlated with GIP but not GLP1, leading to the speculation that suppression of GIP secretion is the main contributor to the observed body weight reduction. Mikada et al. [6] also demonstrated that miglitol decreased plasma GIP levels and increased plasma GLP1 levels; however, it was not clear whether the change in GIP and GLP1 levels was associated with the degree of obesity.

### Basic research of miglitol’s anti-obesity effect and potential mechanisms

There have been five basic research reports including ours [15] demonstrating that miglitol reduces obesity in rodents [16–19]. Furthermore, in support of the results of the clinical studies indicated above with respect to the role of incretin hormones, we measured the blood concentrations of GIP and GLP1 in mice treated with and without miglitol under a high-fat diet, and found no significant difference between the groups (Table 2) [15]. Hamada et al. [17] and Sasaki et al. [18] demonstrated that the blood concentration of GLP1 was significantly higher in the miglitol-treated group than in the untreated group fed a high-fat diet. However, because there was no difference in food intake between the miglitol-treated and untreated groups, it appears that GLP1 did not directly contribute to obesity reduction in these studies. Therefore, despite several lines of evidence demonstrating that miglitol regulates incretin hormones, there is no conclusive evidence that this regulation contributes to obesity reduction.

**Table 2:** Metabolic parameters in 8-week-old mice (Based on Sugimoto et al. [15].)

	*n*	NC	NCM	HF	HFM
Body weight (g)	10–11	21.5 ± 0.2	22.2 ± 0.2	27.3 ± 0.4 ^*, **^	25.8 ± 0.4 ^*, **, ***^
HOMA-R	5	1.4 ± 0.3	1.1 ± 0.3	8.4 ± 1.3 ^*, **^	4.0 ± 0.7 ^*, **, ***^
Weight of epididymal white adipose tissue (g)	9–14	0.27 ± 0.02	0.28 ± 0.01	1.1 ± 0.08 ^*, **^	0.85 ± 0.04 ^*, **, ***^
Weight of subcutaneous white adipose tissue (g)	6	0.3 ± 0.03	Not measured	1.5 ± 0.15 ^*^	0.98 ± 0.12 ^*, ***^
Active glucose-dependent insulinotropic peptide (GIP) (pg/mL)	9–15	28.2 ± 3.6	20.4 ± 2.5	38.8 ± 4.7 ^**^	32.0 ± 4.3
Active glucagon-like peptide 1 (GLP1) (pg/mL)	8–9	54.8 ± 7.9	61.1 ± 4.9	66 ± 7.5	76.9 ± 14.4
Concentration of miglitol (μmol/L)	3–4	Not measured	0.06 ± 0.02	Not Measured	0.26 ± 0.13

^*^ *p* < 0.05 vs NC; ^**^ *p* < 0.05 vs NCM; ^***^ *p* < 0.05 vs HF

Values are means ± SE for 3–15 mice. Four-week-old male C57BL/6 J mice were divided into 4 groups: a control group (NC), which was fed normal chow; a normal chow plus miglitol (NCM) group, which was fed the normal chow plus miglitol; a high fat (HF) group, which was fed the high fat diet; and a high fat plus miglitol (HFM) group, which was fed the high fat diet plus miglitol. At 8 weeks the samples were collected under fasting conditions. Repeated-measures analysis of variance (ANOVA) with Tukey-Kramer post-hoc comparisons were performed for multiple comparisons

The first study on the effects of miglitol in an animal model was reported by Debouno et al. in 1993 [16]. They used SHR/Ntul-cp rats as a model, which show a tendency toward early-onset obesity and non-insulin dependent diabetes mellitus. In this study, miglitol was found to reduce body weight gain without affecting food intake. The other four studies also demonstrated that miglitol reduced body weight gain with no difference of food intake in obese rodent models [15, 17–19]. Our group and Sasaki et al. demonstrated that the reduction of body weight was accompanied by a reduction in epididymal white adipose tissue (WAT) in high-fat diet-induced obese mice (Table 2) [15, 18]. Miglitol also reduced subcutaneous WAT weight in our experiment [15]. In addition, similar to Hamada et al. [17], we further demonstrated that miglitol reduced HOMA-R (Table 2) [15]. Hamada et al. [17] also found that miglitol decreased blood glucose and insulin levels during an oral glucose tolerance test, and reduced blood glucose during the insulin tolerance test, which suggest that miglitol ameliorates insulin resistance, glucose tolerance, and insulin sensitivity in a spontaneous-onset obesity type 2 diabetic mouse model. Shrivastava et al. [19] demonstrated that miglitol improved the plasma lipid profile levels, including T-Cho, triglycerides (TG), phospholipids, free fatty acids, LDL, and very low-density lipoprotein, in high-fat diet-fed rats, and decreased plasma levels of T-cho and TG in hyperlipidemic rats. Based on these reports it is clear that miglitol has an anti-obesity effect in rodents as well as in humans. Sasaki et al. [18] demonstrated that there was no effect of miglitol on locomotor activity, which suggested that an increase in exercise did not mediate the anti-obesity effect induced by miglitol. Common adverse effects of miglitol, such as abdominal distension, diarrhea, or anorexia, were not observed in neither our study [15], the Sasaki et al. [18] study, nor the Debouno et al. [16] study (the other two reports did not refer to side effects.). These findings suggest that suppression of energy intake does not contribute to miglitol’s anti-obesity effect.

Although the first study by Debouno et al. did not verify the mechanism by which miglitol reduces obesity, the other four reports attempted to elucidate the mechanism [15, 17–19]. In particular, miglitol appears to mediate weight reduction via its effects on brown adipose tissue (BAT) and, potentially, white adipocytes. Two mechanisms have been proposed for the former effect, whereas one mechanism has been proposed for the latter effect.

#### Miglitol reduces obesity via activating BAT

Three of the four reports [15, 17, 18] suggested that BAT is involved in miglitol’s anti-obesity effect. BAT is generally considered to disappear after infancy. However, recent studies using positron emission tomography/computed tomography have shown that adult humans retain metabolically active BAT. BAT dissipates energy as heat and contributes to enhancement of energy expenditure, and is thus an important target of obesity treatment [20–22]. UCP1 is a key molecular for BAT thermogenesis. UCP1 uncouples adenosine-5′-triphosphate (ATP) synthesis from substrate oxidation in brown adipocytes. When UCP1 is activated, protons freely flow across the inner mitochondrial membrane, which results in the rapid dissipation of chemical energy as heat. The upregulation of UCP1 induces increased energy expenditure, which contributes to the prevention or reduction of obesity [23]. Recently, our group as well as Sasaki et al. clearly showed that miglitol’s anti-obesity effect was attributed to increased energy expenditure by upregulating UCP1 expression of BAT in mice [15, 18]. Consistent with upregulation of UCP1, miglitol increased oxygen consumption, which is an index of basal metabolism, and promoted heat generation of interscapular BAT in our experiment [15]. Although differences were observed with respect to the age of mice and the dosage of miglitol administered, Sasaki et al. [18] demonstrated that miglitol increased oxygen consumption in mice. Furthermore, we and Sasaki et al. demonstrated that the upregulation of UCP1 is attributed to enhancement of β-adrenergic signaling [15, 18]. Hamada et al. [17] also suggested that BAT might be one of miglitol’s targets, although they proposed a different mechanism, in which miglitol modifies BA metabolism.

#### Proposed mechanism I: Miglitol enters the circulation and directly enhances the β3-aderenergic signaling of BAT

β3-adrenergic signaling enhances UCP1 expression through β3-adrenergic receptor (β3AR) and thus plays a role in alleviating obesity [23]. Stimulation of β3AR induces increased cyclic AMP (cAMP) generation and subsequent activation of protein kinase A (PKA). Hormone-sensitive lipase (HSL), p38 α-mitogen-activated protein kinase (p38αMAPK), and peroxisome proliferator-activated receptor gamma coactivator 1α (PGC1α) are downstream molecules of PKA [15, 24]. Activating transcription factor 2 (ATF2) and cAMP response element binding protein (CREB) bind to the *PGC1α* promoter and enhance its transcription [25].

**Fig. 1:**
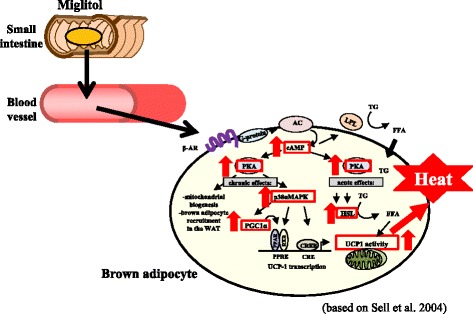
Orally administered miglitol enters the circulation where it directly enhances the β3-aderenergic signaling of brown adipose tissue (BAT). Based on Sugimoto et al. [15], Sasaki et al. [18] and Sell et al. [24]. βAR: beta adrenergic signaling; AC: adenylyl cyclase; cAMP: cyclic adenosine 3′,5′-monophosphate; PKA: protein kinase A; p38αMAPK: p38 α-mitogen-activated protein kinase; PGC1α: peroxisome proliferator-activated receptor gamma coactivator 1α; PPAR: peroxisome proliferator-activated receptor; RXR: retinoid X receptor; PPRE: PPAR response element; CREB: cAMP response element binding protein; CRE: cAMP response element; HSL: hormone-sensitive lipase; UCP1: uncoupling protein 1; LPL: lipoprotein lipase; TG: triglyceride; FFA: free fatty acids

We clarified that miglitol increased the mRNA and protein expression of UCP1 and the expression of the four proteins involved in the signaling cascade (PKA, HSL, p38αMAPK, and PGC1α) in high-fat diet-induced obese mice. Miglitol increased the amount of cAMP and phosphorylated PKA (*p*PKA) protein levels in the presence of a β3AR agonist in our experiment, which confirmed that miglitol enhances β3-aderenergic signaling [15]. Sasaki et al. [18] investigated the direct effect of miglitol on brown adipocytes using HB2 cells (differentiated immortalized brown preadipocytes). Miglitol increased the amount of cAMP and the phosphorylation of CREB and ATF2 under βAR agonist (isoproterenol) stimulation, which further suggests that miglitol directly enhances β-aderenergic signaling. Miglitol was reported to transfer into the blood circulation, unlike other αGIs [26, 27]; indeed, miglitol was detected in the blood in our study (Table 2). Sasaki et al. [18] also showed that intraperitoneal injection of miglitol increased oxygen consumption in mice, which suggests that circulation of miglitol directly activates BAT. Both our study and the Sasaki et al. study [15, 18] suggest that miglitol enters the blood circulation where it directly activates BAT in mice (Fig. 1). Anti-obesity drugs that act on the central nervous system and reduce food intake tend to cause psychological side effects [1, 28]. Therefore, the finding that miglitol directly acts on peripheral tissues (such as BAT) suggests a new and fascinating mechanism for obesity inhibition [18].

#### Proposed mechanism II: Miglitol activates BAT via bile acid (BA)

BA was reported to stimulate the G protein-coupled receptor of BAT (TGR5) and increase energy expenditure by activating type 2 iodothyronine deiodinase (DIO2) [29], a cyclic AMP-dependent thyroid hormone-activating enzyme. The BA-TGR5-cAMP-DIO2 signaling pathway has been reported to induce the expression of UCP1 in BAT [29]. Hamada et al. [17] demonstrated that miglitol increased BA levels in both the feces and portal blood and tended to increase DIO2 expression. There was also a positive correlation between the portal blood BA level and DIO2 gene expression. Based on these results, the authors speculated that enhancement of BA by miglitol would increase the DIO2 expression of BAT, contributing to an increase in UCP1 expression. Although the energy expenditure of mice (e.g., body temperature, oxygen consumption) was not measured in their experiment, their results nonetheless support the theory that activation of BAT by miglitol contributes to its anti-obesity effect.

#### Potential effect of miglitol on white adipocytes

Shrivastava et al. [19] demonstrated that miglitol inhibited the differentiation of 3T3-L1 preadipocytes in a dose-dependent manner. Based on these results, they hypothesized that miglitol’s anti-obesity effect is attributed to prevention of the lipid accumulation of WAT. On the other hand, Sasaki et al. [18] demonstrated that miglitol did not have an effect on white adipocyte differentiation markers, even at a millimolar dose. Therefore, whether or not miglitol has a direct effect on the differentiation of white preadipocytes requires further investigation.

Collectively, based on the evidence accumulated to date, it is clear that miglitol has an anti-obesity effect in rodents as well as humans. Although there are several hypotheses of the mechanism by which miglitol reduces obesity, we consider that BAT activation is one of the crucial mediators of this effect.

### Clinical perspectives

The fact that miglitol has been actively used clinically since 1998 with no severe side effects is noteworthy and shows its potential for applications in obesity treatment, although the exact mechanism has not yet been completely elucidated. Nevertheless, miglitol is associated with minor side effects such as gastrointestinal symptoms [30]. If these side effects are tolerable, miglitol shows promise as an anti-obesity drug. However, if these side effects are not acceptable, further investigations into the mechanism of action of miglitol would be warranted for the development of a new anti-obesity drug.

At present, the U.S. FDA has approved four anti-obesity drugs for short-term use (usually considered less than 12 weeks) and five anti-obesity drugs for long-term use [2]. The short-term use anti-obesity drugs are noradrenergic agents (phentermine, diethylpropion, phendimetrazine, and benzphetamine), and seem to have an equivalent or a more slightly enhanced effect on body weight reduction compared to miglitol [31]. However, these noradrenergic agents have potential for abuse and are also contraindicated in patients suffering from advanced cardiovascular disease, uncontrolled hypertension, and hyperthyroidism [31]. Because miglitol has no risk of dependency and is not contraindicated for many diseases, we believe that it could be more easily tolerated and administered than these other drugs. The long-term use anti-obesity drugs are orlistat (a gastrointestinal lipase inhibitor), lorcaserin (a serotonin 2C receptor agonist), phentermine/topiramate extended-release (a sympathomimetic amine with anoretic effect/mechanism unknown), naltrexone/bupropion (an opioid receptor antagonist/aminoketone antidepressant), and liraglutide (a GLP1 receptor agonist). Because the lengths of the clinical studies conducted thus far to examine miglitol’s anti-obesity effect are shorter than those conducted for these five FDA-approved anti-obesity drugs [2, 31], we cannot directly compare the efficacy for body weight reduction between miglitol and these other drugs. The FDA approved orlistat as an anti-obesity drug in 1999, and it has been used clinically ever since. Although orlistat is generally as well tolerated as miglitol, it is contraindicated in patients with chronic malabsorption syndrome and cholestasis. Furthermore, it is recommended that patients using orlistat supplement their vitamin intake [2]. Therefore, we believe that miglitol would be easier to use than orlistat for the general population. Since the other four anti-obesity drugs were approved only a few years ago [2], sufficient evidence of their safety is not yet available.

The FDA-approved anti-obesity drugs described above generally all function via inhibiting energy intake; therefore, they fall into the general category of appetite suppressants and lipid absorption inhibitors [2]. Development of a drug that could enhance energy expenditure would provide a new category of anti-obesity drug altogether. Our previous report and that of Sasaki et al. support the opinion that miglitol activates BAT and induces upregulation of energy expenditure [15, 18]. The work of Hamada et al. [17] also supports this possibility, although their proposed mechanism is different. Nevertheless, these reports collectively suggest that miglitol activates BAT, at least in mice. There has been no clinical investigation of energy expenditure (i.e., basal metabolism or body temperature) in response to miglitol administration. Such detailed evaluations in future clinical trials could help to verify whether BAT is involved in miglitol’s anti-obesity effect.

In Japan, a clinical trial of miglitol’s effect on patients with pediatric type 1 and 2 diabetes is ongoing to evaluate its potential as an anti-diabetic drug. If this trial demonstrates that miglitol is safe for children, the efficacy of miglitol’s anti-obesity effect on pediatric obesity would likely be investigated further in the future. Since aging leads to loss of BAT [21], miglitol’s anti-obesity effect might be stronger in children as compared with that observed in adults.

## Conclusions

Evidence of miglitol’s anti-obesity effects has been gradually accumulating in the field of obesity research. Several clinical and basic research studies have clearly shown the anti-obesity effect of miglitol in humans and rodents. Although further research is required to elucidate the mechanism of this effect, we can now conclusively state that miglitol shows good potential as a novel agent for obesity treatment.

## Abbreviations

αGI: Alpha-glucosidase inhibitor
BAT: Brown adipose tissue
HOMA-R: Homeostasis model assessment of insulin resistance
T-Cho: Total cholesterol
LDL: Low-density lipoprotein
GIP: Glucose-dependent insulinotropic peptide
GLP1: Glucagon-like peptide 1
UCP1: Uncoupling protein 1
β3AR: β3-adrenergic receptor
PKA: Protein kinase A
HSL: Hormone-sensitive lipase
p38αMAPK: p38 α-mitogen-activated protein kinase
PGC1α: Peroxisome proliferator-activated receptor gamma coactivator 1α
ATF2: Activating transcription factor 2
CREB: cAMP response element binding protein
DIO2: type 2 iodothyronine deiodinase
